# Flashback behavior and safety implications of hydrogen–natural gas mixtures

**DOI:** 10.1038/s41598-025-22435-y

**Published:** 2025-11-12

**Authors:** Radovan Nosek, Marek Patsch, Peter Pilát, Branislav Zvada, Alexander Backa

**Affiliations:** 1 Department of Power Engineering, Faculty of Mechanical Engineering, University of Žilina, Univerzitná 8215/1, Žilina, 010 26 Slovakia; 2 Research Centre, University of Žilina, Univerzitná 8215/1, Žilina, 010 26 Slovakia

**Keywords:** Safety combustion, Hydrogen, Natural gas, Quenching distance, Mekker-Fischer burner, Energy science and technology, Engineering

## Abstract

Hydrogen blending in natural gas systems is a key transitional strategy for reducing carbon emissions. This study explores the influence of hydrogen on combustion properties, including flame flashback risk, quenching distance, and energy efficiency. Experimental and computational analyses demonstrate that hydrogen addition increases flame speed but reduces calorific value and quenching distance, thereby impacting combustion stability and safety. Findings suggest that optimizing burner design and combustion control strategies is essential for safely and efficiently using hydrogen-enriched natural gas. Experimental validation confirmed that a 1.50 mm channel dimension effectively prevented flame flashback for hydrogen concentrations up to 40% in natural gas. As energy systems evolve toward decarbonization, this research provides critical insights into the feasibility and challenges of hydrogen integration in residential or industrial applications. The study investigated the combustion behavior of natural gas enriched with various concentrations of hydrogen (up to 25%). Dynamic or fluctuating mixing conditions were excluded, as the implementation of such a system in energy sector applications would necessitate a stable and well-defined gas composition.

## Introduction

The anthropogenic impact on the environment is increasingly evident these days. Reducing this impact and influencing the ongoing climate change can be achieved mainly by reducing greenhouse gas emissions, which leads to increased interest in decarbonization strategies in various industrial sectors, including heating and energetics. Mitigating climate change through the reduction of greenhouse gas emissions necessitates the transition from fossil fuels, which have a substantial carbon footprint, to renewable and sustainable alternatives. Member States of the European Union (EU) have pledged to achieve climate neutrality by 2050. The transition to carbon-free technologies requires gradual decarbonization of existing infrastructure. One of the promising transitional strategies for the gradual decarbonization of energetics, based on the use of natural gas, is its enrichment with hydrogen (H_2_)^1,2^. A mixture of natural gas enriched with hydrogen to a certain concentration can be transported through existing pipeline networks; due to the possible risk of hydrogen corrosion, several studies are devoted to the issue^3,4^.

Boilers, widely used in industrial and residential applications, play a key role in heating, heat recovery, and steam generation. The transition to hydrogen-enriched fuels in these systems presents opportunities but also challenges. Changing the fuel, in this case, a mixture of natural gas and hydrogen, affects both the performance and safety parameters of the device.

Replacing the original fossil fuel with this mixture, which has a different calorific value and other physicochemical properties, affects the entire combustion process. A mixture of natural gas enriched with hydrogen in various ratios has a heat value by volume that is influenced by the percentage of individual components in the mixture. The heat value by volume, i.e., the amount of heat released during the combustion reaction, can be determined either computationally, as shown in this article, or experimentally, primarily using a Junkers calorimeter. For the computational determination of the heat value by volume, values from chemical tables are used and calculations are performed for different hydrogen concentrations in the mixture. The calculations show a decrease in the heat value by volume of the mixture as the proportion of hydrogen increases. This is because hydrogen has a significantly lower calorific value per cubic meter. At a 20% volume concentration of hydrogen in the mixture, the heat value by volume decreases by 14%. Several authors have investigated the experimental evaluation of the heat of combustion of a natural gas–hydrogen mixture and the thermal output achieved during combustion. Sorgulu et al. used a simple experimental method to compare the achieved thermal output of a heating device^5^. The results of this study show that a mixture of 20% hydrogen and 80% natural gas yields an 8% saving of natural gas (in volume percent), although there was a 15.9% increase in heating time.

One of the key parameters affecting boiler performance and operation safety is the laminar flame velocity of the fuel mixture^6^. Laminar flame velocity is a fundamental property of combustible mixtures, representing the speed at which the flame front propagates through a quiescent fuel-air mixture. It plays a key role in understanding and predicting combustion behavior, including flame stability, flashback tendency, and overall combustion efficiency^7,8^. In the context of boilers, changes in laminar flame velocity due to hydrogen addition can significantly affect burner design, flame stability, emissions and operational safety. Several experimental and simulation-oriented studies, e.g. Burbano et al. confirm the favorable environmental impact of adding hydrogen to natural gas, which will be reflected in the reduction of CO_2_ emissions due to the absence of carbon in the fuel^9^. New natural gas boilers mostly meet the performance and safety conditions, they are so-called H_2_-ready (these are condensing boilers that can burn a natural gas mixture enriched with hydrogen in a limited proportion, most often up to 20% by volume, their operational resistance to flame flashback is achieved by using a mesh screen on which the combustion mixture burns). The use of this fuel mixture of natural gas with hydrogen in older, existing devices, for which safety risks are known from technical practice, is problematic. Zhan et al. evaluated the effects of adding 0 to 40% hydrogen (in volume percent) on emissions, flame shape, and burner temperature of a water heater at a thermal load of 0.7–2.3 kW^10^. The internal flame height first increased and then decreased with the increasing H_2_ content in the mixture, while the burner temperature changed in the opposite direction – increasing instead. The maximum inner flame height and the minimum temperature of the burner both appear at the hydrogen blending ratio of 10–20%. The findings of the study showed that a hydrogen content of 40% was the maximum that could still guarantee stable operation (this working condition causing the material surface overheated and turn red, which brings potential safety hazards to combustion). In this case, the burner operation remained safe at a constant thermal load.

Several experimental and numerical studies have provided valuable insights into the effects of hydrogen addition on the laminar flame velocity of a methane–hydrogen gas mixture. Hu et al. conducted an experimental and numerical study of the laminar combustion characteristics of methane-hydrogen-air premixed flames^11^. They observed that the addition of hydrogen led to an increase in the laminar combustion rate, with the effect becoming more pronounced at higher hydrogen fractions. Salzano et al. also studied the behavior of hydrogen–methane mixtures and experimentally confirmed an increase in the laminar burning rate and the rate of pressure rise, depending on the chemical composition of the fuel^2^. The above-mentioned scientific studies lead to an understanding of the issues of flashback and quenching distance, which have a fundamental impact on the safety of combustion device operation.

Previous studies have investigated the quenching distance of methane-hydrogen mixtures with air, confirming that the quenching distance is a function of the percentage of hydrogen in the methane-hydrogen mixture, the excess air ratio and the pressure. Most studies have focused on the combustion of such mixtures in piston internal combustion engines and have not considered combustion in heat sources. It has been observed that the flame quenching phenomenon has a probability nature at the region where the distance between the two discs approaches the quenching distance^12^. A comprehensive summary of recent research progress on internal combustion piston engines burning hydrogen-enriched compressed natural gas (discontinuous combustion) is provided in the study by Yan et al.^13^. The conclusions derived in their study are also applicable to heat sources (continuous combustion), particularly in terms of performance, efficiency, combustion, and emission characteristics.

Jung et al. conducted an experimental verification of the flame propagation characteristics of a hydrogen–air mixture using an improved annular stepwise diverging tube^14^. They also used an infrared detection camera to visualize the flame behavior. Their study provided a foundation for our experimental assessment of the quenching distance of a natural gas–hydrogen–air mixture in heat sources. The conditions for the safe operation of a heat source in terms of quenching distance are described, evaluated through numerical calculations, simulations, and experimental measurements.

The study by Semenikhin et al. provides a brief overview of kinetic models for the combustion of hydrogen and methane-hydrogen mixtures^15^. Nine kinetic models (three Konnov-2019 models; two Stagni models; CRECK-2020; Wang-2018; NUIGMech1.0; and GRI Mech 3.0) were studied for numerical simulation of nitrogen oxide concentrations during the combustion of methane and hydrogen in combustion chambers. The predictions of these models were then rigorously verified against experimental data from leading world laboratories. The results and conclusions of this study were subsequently used to select the optimal computational tool for performing the modeling of the combustion of natural gas and hydrogen mixtures.

Based on the reviewed studies, it is evident that hydrogen addition significantly affects key combustion parameters such as flame velocity, flashback tendency, and quenching distance, all of which directly influence the safety of gas boiler operation. To address these aspects in practical applications, an experimental and computational methodology was designed and is described in the following section.

## Methodology

**Fig. 1:**
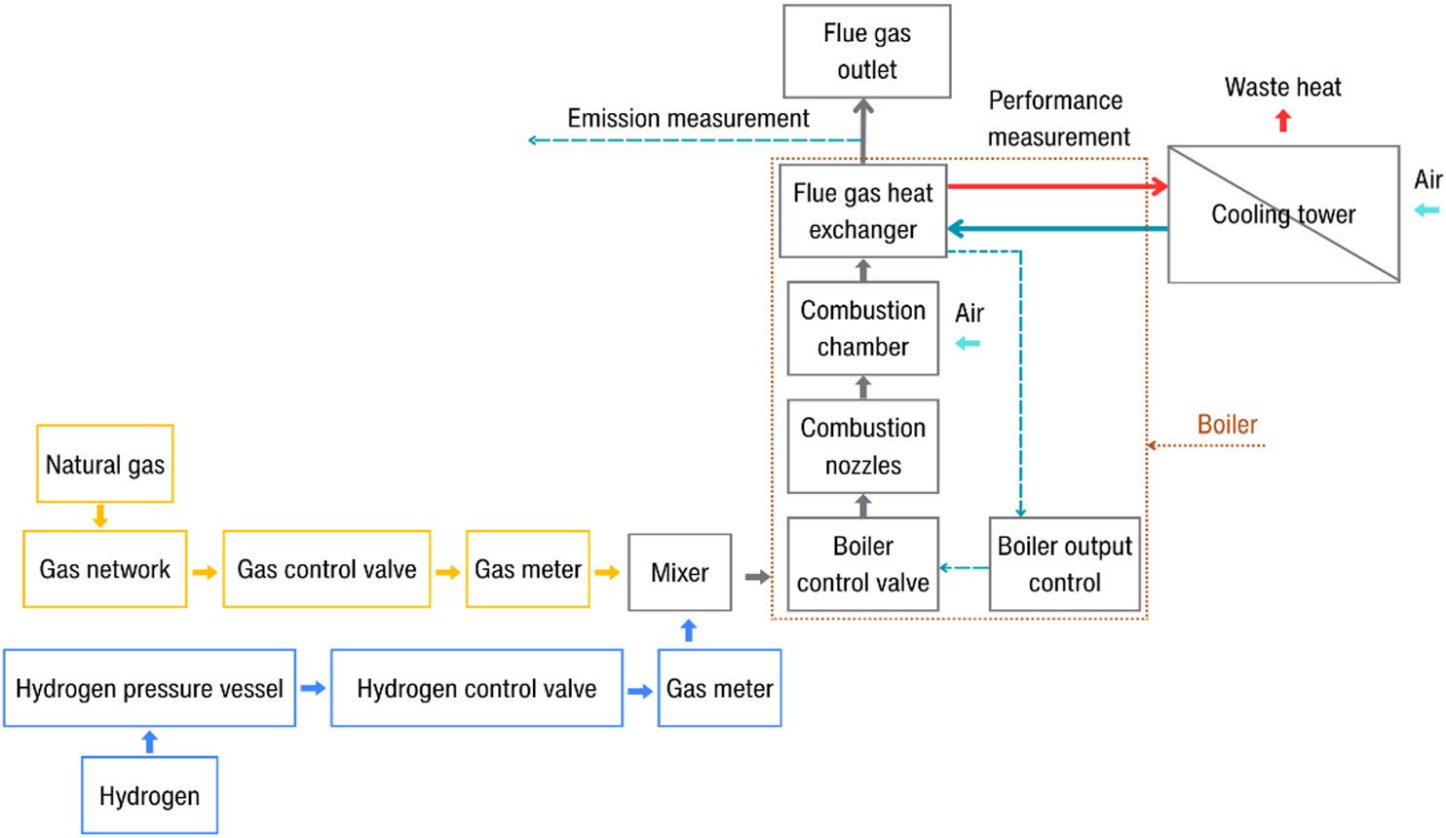
The scheme of experimental setup.

The measurements were conducted on a Mekker-Fischer burner. In addition, an atmospheric gas boiler Viessmann Vitopend 100 with a nominal output of 29 kW, equipped with a commonly used type of atmospheric burner prevalent under local conditions, was considered as a representative system for assessing possible modifications. The Mekker-Fischer burner was used to visualize experiments and identify the optimal variant with the aim of achieving a conversion that would be as simple and cost-effective as possible in practical applications. The combustion unit works by supplying air with a fan and fuel through a gas valve, mixing them in the burner chamber, and igniting the mixture with a spark electrode while the ionization electrode monitors the flame. The flame heats the heat exchanger, transferring energy to the water circuit. This type of burner could pose safety concerns when hydrogen is blended with natural gas. The boiler was connected to a gas supply line delivering a mixture of natural gas and hydrogen (Fig. 1).

Hydrogen was supplied from a high-pressure cylinder at 160 bar. Pressure regulators (GCE ProControl, ISO 5171) were used to reduce and stabilize the pressure of a gases from a high-pressure source to a lower, usable outlet pressure in the range of 0–16 bar, with an accuracy of ± 0.1 bar. A pressure control valve ensured stable operation.

**Fig. 2:**
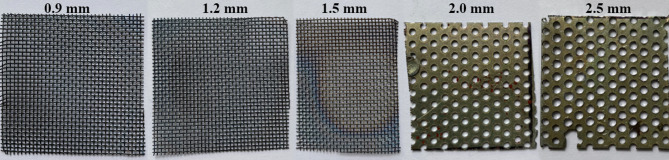
Images of wire mesh screens with varying pore sizes for quenching distance experiments.

The correct mixing ratio of the components was controlled using a calibrated volumetric flow meter (Type PREMAGAS; G6 – MKM/BK) with thermal compensation. The maximum flow rate of gas meter is 10 m^3^/h and permissible error is ± 3%. A stabilization pipe with integrated pressure and temperature sensors was installed upstream of the flow meter. The mixing of natural gas and hydrogen was carried out in a T-junction equipped with a turbulence-inducing mesh to ensure thorough mixing and to prevent separation of the individual components. The calculated quenching distance values were experimentally verified using a Mekker-Fischer type laboratory burner. A series of wire mesh screens with systematically varied pore sizes were utilized (Fig. 2). Wire screens consisted of woven stainless-steel meshes with nominal pore diameters of 0.9, 1.2 and 1.5 mm, and perforated steel sheets with circular apertures of 2.0 and 2.5 mm.

The thermal output from the boiler was directed through a certified test rig for boiler certification, allowing for accurate measurement of performance characteristics. The excess heat was dissipated in dry coolers and a cooling tower. Emission measurements in the flue gas were carried out using the MADUR PHOTON, which enables the detection of CO and NO_x_ emissions in the range of 1–3000 ppm with an accuracy of ± 3 ppm abs. or 3% relative. The influence of varying hydrogen concentrations on emission parameters is beyond the scope of this study; consequently, these results are not presented. This topic will be addressed in a subsequent publication.

Temperature measurements within the combustion chamber were performed using NiCr-Ni (type K) thermocouples, with a temperature range from − 200 °C to 1370 °C and a measurement accuracy of ± 0.1 K. All thermocouples were calibrated prior to use.

Data acquisition was carried out using an Almemo Ahlborn 5690-2 M data logger, with direct export of the measured data to Excel.

While experimental studies are crucial, computational simulations offer a complementary approach that allows for the investigation of parameters across a broader range and provides insight into fundamental physicochemical processes^16^. Computational simulations of natural gas and hydrogen mixture combustion are increasingly recognized as a crucial tool for predicting the performance of existing energy systems. This approach offers the advantage of evaluating system behavior without the necessity for extensive experimental validation for each specific device, which can vary significantly based on manufacturer, model, and production year. A range of computational simulation software packages are available, including general-purpose tools and those specifically tailored for combustion analysis. The accuracy of these simulations is primarily dependent on the selected computational model and the input data. Among these tools, Cantera, an open-source, object-oriented software library designed for problems involving chemical kinetics, thermodynamics, and transport processes, has become a widely adopted platform for such simulations^17^.

In this study, a one-dimensional flame solver was applied. As the combustion mechanism in this solution instance, the GRI-Mech 3.0 mechanism was applied^18^. It is a widely used detailed kinetic mechanism for natural gas combustion and is applicable at pressures where the ideal gas law holds. It includes nitric species for NO_x_ predictions with 53 species and 325 reactions. A basic representation of pipeline gas in Slovakia was employed for flame velocity analysis.

## Results: modelling and experiments

Simulations explored hydrogen volume fractions ranging from 0% to 25%. A 25% hydrogen limit was chosen as it already represents a high concentration that significantly impacts combustion. Heat sources currently capable of operating with mixtures of H_2_ and natural gas (referred to as H₂-ready) are generally adapted for the problem-free use of up to 20% hydrogen, due to the guaranteed safety of the combustion equipment and pipeline system. The Hydrogen Boiler Feasibility Study (Eunomia) reports that most existing gas boilers can safely operate with hydrogen admixture levels up to 20% by volume, with trials showing no performance or safety issues at this concentration, which is also regarded by manufacturers as the maximum recommended limit for domestic applications^19^. Therefore, 25% H_2_ was considered in this work as a limiting concentration that reflects practical constraints of burner design while still allowing investigation of the impact on combustion properties.

Lambda (λ) was adjusted between 0.5 and 2.2. All modeling activities occurred under atmospheric pressure (1 atm), starting at a temperature of 300 K, utilizing data drawn from Table 1.

The change in mixture properties was determined using calculations based on the relations specified in the ISO 6976:2016 standard^20^. The lambda value of a combustion mixture in a combustion mixture plays a key role in flame speed, thermal characteristics, and species distribution. Lean mixtures exhibit lower flame speeds than stoichiometric ones because less heat is released, despite higher flame temperatures. Under stoichiometric conditions, flame propagation and heat release are maximized. Rich mixtures also show a decline in flame velocity as excess fuel reduces heat generation and flame temperature^21^.

**Table 1:** Natural gas composition for calculation: comparison of 0% and 25% hydrogen content^22^.

Components of natural gas	Chemical formula	Molar composition of the mixture at 0% hydrogen concentration [mol]	Molar composition of the mixture at 25% hydrogen concentration [mol]
Methane	CH_4_	0.92488	0.69365
Ethane	C_2_H_6_	0.05472	0.04104
Propane	C_3_H_8_	0.02039	0.01529
Hydrogen	H_2_	0	0.25

**Fig. 3:**
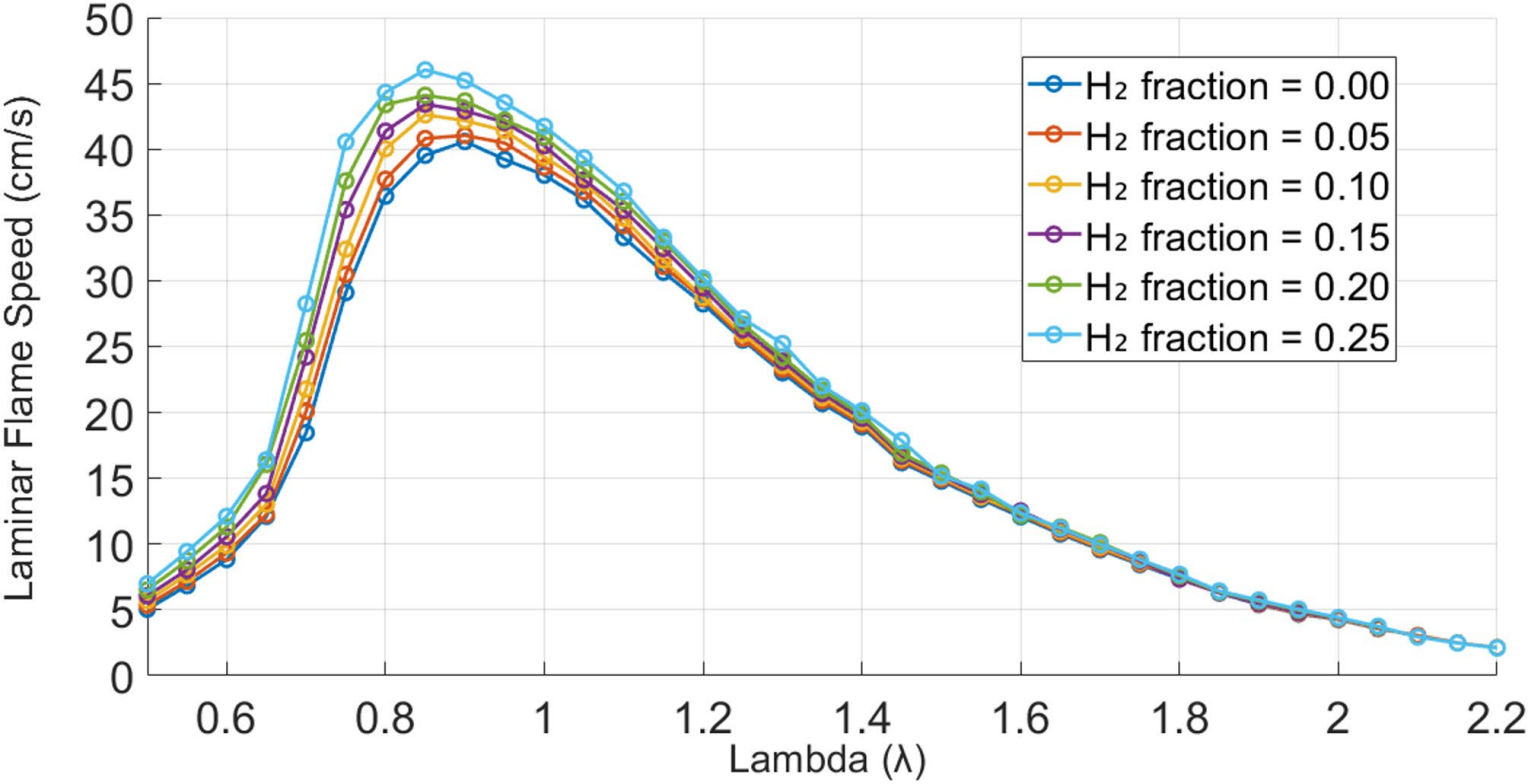
Laminar flame speed vs. lambda (λ) for different hydrogen fractions.

Figure 3 shows computed laminar flame speeds for various hydrogen fractions. The flame speed increases with hydrogen concentration, particularly near stoichiometric conditions (λ ≈ 1), while at higher λ values the effect of hydrogen addition becomes less significant. At sufficiently lean conditions, the curves nearly coincide, indicating that hydrogen has little influence on flame speed. Hydrogen’s high diffusivity enhances fuel-oxidizer mixing, while its higher flame temperature increases thermal energy release, collectively accelerating flame propagation. At λ = 0.5, the flame speed increases from 5.1 cm/s for pure methane (0% H_2_) to 6.98 cm/s at 25% H_2_ (37% increase). At λ = 0.7, it rises from 18.6 to 28.24 cm/s (52%), and at λ = 1.0 from 38.03 to 41.73 cm/s (10%). For pure methane, the peak occurs at λ ≈ 0.91, shifting to λ ≈ 0.83 at 25% H_2_.

As hydrogen increases, the peak flame speed shifts to richer mixtures due to higher flame temperature, faster reaction rates, and improved fuel utilization. For pure methane, the peak occurs at λ ≈ 0.91, shifting to λ ≈ 0.83 at 25% hydrogen.

These results imply that conventional burner designs optimized for methane may no longer ensure stable combustion when operated with hydrogen-enriched gas. The increased flame speed and shift in peak propagation conditions may require adaptive control systems or pre-mixing strategies to maintain safety and efficiency across a range of hydrogen concentrations. In practical terms, the adjustment of burner port dimensions or implementation of swirl stabilization may be necessary, particularly in systems not originally designed for high hydrogen content.

### Flame flashback

Flame flashback is a critical safety and operational issue in premixed combustion systems, characterized by the undesired upstream propagation of the flame from the combustion zone into the burner and premixing ducts. This phenomenon occurs when the local flame propagation speed exceeds the velocity of the incoming fresh gas mixture. In burner nozzles it is particularly pronounced in the boundary layer adjacent to the walls, where viscous effects reduce the gas velocity; if the flame speed in this boundary layer surpasses the local flow velocity, the flame can anchor to the wall and propagate upstream into the burner ports.

The addition of hydrogen to natural gas substantially increases the propensity for flashback because of several fundamental physical and chemical differences. Hydrogen has a much higher laminar flame speed—under stoichiometric conditions, the laminar flame speed of pure hydrogen is roughly six times that of methane — which makes the flame more reactive and more able to outpace the bulk flow in regions of reduced velocity. Hydrogen also exhibits a lower minimum ignition energy and a smaller quenching distance compared with typical natural gas blends; the reduced quenching distance allows flames to remain stabilized closer to solid surfaces, increasing the risk of anchoring at or migrating into burner openings. In addition, hydrogen’s high molecular diffusivity modifies the flame structure through preferential diffusion: the lighter hydrogen molecules tend to diffuse more rapidly toward the flame front, which can locally enrich the mixture (decrease the local λ value) and thereby raise the local flame speed, further promoting flashback.

When hydrogen is blended with natural gas, changes in both exit velocities and flame propagation behavior make flashback a major safety concern. The higher reactivity and reduced quenching distance associated with hydrogen make it more likely that the flame will propagate upstream, especially under transient conditions such as power regulation or changes in operating load. These tendencies have been observed experimentally and numerically in several studies. For example, Zhao et al. reported flashback in a cooking burner and in a gas oven at a hydrogen blending ratio of 20% by volume during power regulation, when the flame moved back into the burner body^23^. Lo Basso et al. reached similar conclusions for a condensing boiler, confirming that hydrogen blending increases flashback risk^24^. Advanced numerical studies that include conjugate heat transfer, such as those by Fruzza et al., show that thermal feedback and preferential diffusion effects at slit ends or plate geometries can significantly enhance flashback velocities, highlighting the complex interplay of aerodynamic, thermal and chemical effects that control flashback in practical burner designs^25^.

### Quenching distance

Flame quenching occurs when the heat loss from the flame exceeds the heat generated by the combustion reaction, preventing the chemical reaction from continuing due to the inability to reach the activation energy of molecules. Fukuda et al. conducted a detailed study on the effect of hydrogen addition on the quenching distance of methane-hydrogen mixtures^12^. Their results revealed a notable decrease in quenching distance with increasing hydrogen content, especially in lean and stoichiometric conditions. For example, at 9.1% hydrogen concentration, the quenching distance decreased by 6% for stoichiometric (λ = 1.0) and 15% for lean (λ = 1.6) mixtures. At 23.1% hydrogen, these reductions were 15% and 36%, respectively.

The quenching distance parameter represents the minimum tube diameter that allows premixed flame propagation and is crucial for flashback prevention. The equation for calculating quenching distances incorporates parameters related to heat transfer, reaction kinetics, and gas properties, following the classical formulation of Potter and Berlad^26^.

1 $$d = \sqrt {\frac{{2\kappa _{r} \kappa _{F} FG_{i} \left( {\frac{{n_{1} }}{{n_{2} }}} \right)^{m} \left( {\frac{{\kappa RT}}{{DC_{p} P}}} \right)_{F}^{m} \bar{W}}}{{C_{{p,r}} \;\overline{{C_{{p,F}} }} W}}}$$

**Fig. 4:**
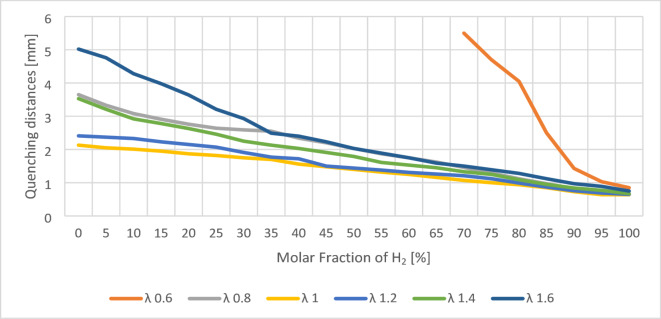
Graph of calculated quenching distances for different λ ratios and hydrogen concentrations.

The thermal capacity (*C*_*p, r*_) and thermal conductivity (*κ*_*r*_) determine heat transfer in the reaction zone. Combustion dynamics are influenced by the reaction rate (*w*) and the molar fraction of fuel ( *X*_*f*_), which are reflected in the averaged reaction term ($\:\bar{W}$) and the stoichiometric mole ratio (*n*_*1*_*/n*_*2*_)^*m*^. The characteristic channel dimension (*d*) is the quenching distance, with the geometry coefficient (*G*_*i*_) accounting for channel effects. Mass transfer is governed by the diffusion coefficient (*D*), while pressure (*P*) affects reaction kinetics. The mean specific heat of the fuel ($\: \overline{{C_{{p,F}} }}$) describes mixture properties. The gas constant (R) and temperature (T) define the thermodynamic state, and a constant (*F*) expresses the heat required to sustain the flame. Using Eq. (1), quenching distances were calculated for specific hydrogen concentrations and *λ* values, providing insights into flame stability (Fig. 4).

The smallest characteristic channel dimension occurs at the stoichiometric mixture ratio and the highest hydrogen concentration. Additionally, hydrogen has a pronounced effect on the combustion of lean mixtures (λ = 1.6), where this influence is most significant. However, during the combustion of pure hydrogen, λ has a weaker impact compared to natural gas. The sharp decrease in quenching distance for rich mixtures is due to a shift in flammability limits. Model results indicate that hydrogen significantly reduces the quenching distance, increasing the risk of flame flashback into the burner during boiler operation. A hydrogen-enriched mixture has higher kinetic energy, enhancing the ejector effect and increasing air entrainment into the burner. This shifts the mixture from rich to stoichiometric, reducing the required characteristic channel dimension for the quenching distance. Additionally, the quenching distance is highly sensitive to hydrogen concentration in the mixture. Due to the complexity of determining precise λ ratios across various gas systems, it is recommended to modify these devices to ensure the characteristic channel dimension meets the safety requirements for a predefined hydrogen content in natural gas. Given the potential for higher hydrogen concentrations in natural gas, a 1.50 mm characteristic channel dimension is recommended to ensure resistance to flame flashback, even at 40% hydrogen content. Compared to other gaseous fuels, hydrogen exhibits the smallest quenching distance. In real-world systems, the variation of hydrogen concentration due to supply network fluctuations or operational blending can cause dynamic shifts in quenching behavior, making real-time monitoring or design adjustments essential.

### Gross calorific value

The gross calorific value expresses the amount of heat released during the chemical reaction of combustion, specifically the complete burning of a fuel. This value is determined when all combustion products are brought back to the initial temperature of the reactants - fuel and oxidizer and water vapor condensation occurs. Therefore, the gross calorific value of a gas represents the amount of heat released by burning a unit volume of the fuel.

For the gross calorific value of an ideal gas on a molar basis, the following applies:

2 $$\:{{(H}_{c})}_{G}\left({t}_{1}\right)={{(H}_{c})}_{G}^{O}\left({t}_{1}\right)=\sum\:\text{}{{xi\bullet\:\left[\right(Hc]}_{G}^{0}]}_{i}\:\left({t}_{1}\right)\text{}$$

For the gross calorific value of a real gas on a volumetric basis, the following applies:

3 $$\:{\left({H}_{v}\right)}_{G}\left({t}_{1};{t}_{2};{p}_{2}\right)=\frac{{\left({H}_{c}\right)}_{G}^{O}\:\left({t}_{1}\right)}{V}$$

The molar volume of a real gas *V* is calculated using the following equation:

4 $$\:V=Z({t}_{2},{p}_{2}) \cdot \:R\bullet\:\frac{{T}_{2}}{{p}_{2}}$$

The gross calorific value of a real gas on a volumetric basis, denoted as $\:{\left({H}_{v}\right)}_{G}\left({t}_{1};{t}_{2};{p}_{2}\right)$ is expressed in megajoules per cubic meter. The gross calorific value on a molar basis for an individual component in the state of an ideal gas is represented as $\:{\left[{{(H}_{c})}_{G}^{O}\right]}_{i}$ and measured in kilojoules per mole. Similarly, $\:{{(H}_{c})}_{G}^{O}\left({t}_{1}\right)$ refers to the gross calorific value on a molar basis for the gas mixture of natural gas and hydrogen in the state of an ideal gas, also given in kJ/mol. In contrast, $\:{{(H}_{c})}_{G}{t}_{1}$ represents the gross calorific value of the same gas mixture in the state of a real gas. Finally, the real molar volume of the gas in the mixture, denoted as *V*, is measured in cubic meters per mole.

**Fig. 5:**
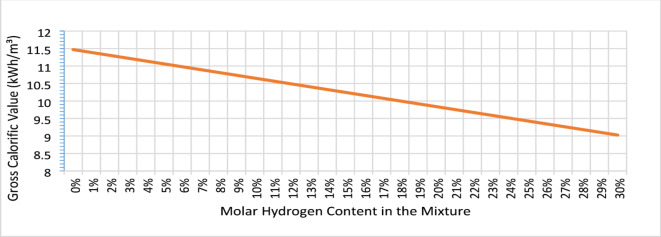
Effect of hydrogen content in the mixture on the gross calorific value according to the GERG-2008 Model.

With an increase in hydrogen content in the mixture, a significant change in the gross calorific value of the mixture can be observed (Fig. 5). This is due to the fact that hydrogen has a considerably lower calorific value per cubic meter, or volumetric heating value. At a hydrogen concentration of 20% by volume, the gross calorific value decreases from 11.46 kWh/m^3^ to 9.83 kWh/m^3^, representing a 14% reduction. This decrease in energy density suggests that boiler control systems may require recalibration or modification to account for reduced heating output per unit volume. Additionally, gas billing mechanisms based on volumetric energy delivery might need adjustment to reflect lower gross calorific values in hydrogen-enriched gas.

The GERG-2008 equation of state is a wide-range reference equation of state based on current state-of-the-art models, used for natural gases and similar mixtures. It is based on a multi-fluid mixture model and is explicitly expressed in terms of reduced Helmholtz energy, adopted as an ISO standard. It has been used to calculate physical properties of natural gas and hydrogen mixtures, such as density, heat value by volume, which affect the operation of natural gas heat sources.

However, beyond the purely energetic considerations, it is crucial to address the safety of operating these mixtures. Small concentrations of hydrogen in the mixture do not have a significant impact on the safety of gas boiler operation, they will only be reflected in a small change in the heating value of the mixture compared to pure natural gas. Increasing the hydrogen concentration affects the safety of operation to a dangerous level, but by adding a suitable mesh, operation becomes safe again.

### Experimental confirmation of quenching distance

The results confirm that at a channel dimension of 1.50 mm, the flame is reliably quenched, preventing its propagation into the burner space. Part of the experiment also focused on determining the quenching distance for a 20% hydrogen concentration in natural gas. These findings indicate that a channel dimension of 2.50 mm is sufficient to prevent flame flashback; however, this outcome is largely influenced by the specific setup, in which the flame burns in a fuel-rich region and a significant portion of the required air is supplied by diffusion from the surrounding environment. Consequently, this value cannot be regarded as universally applicable to all types of equipment, as combustion conditions may vary depending on the λ (lambda) ratio.

The experiment further demonstrated that the channel length—i.e., the thickness of the screen—has little influence on flame quenching. A screen thickness of only 1 mm reliably prevented flame propagation at a 20% hydrogen concentration. Moreover, the material between the openings exhibited only a minor effect on the quenching process.

**Fig. 6:**
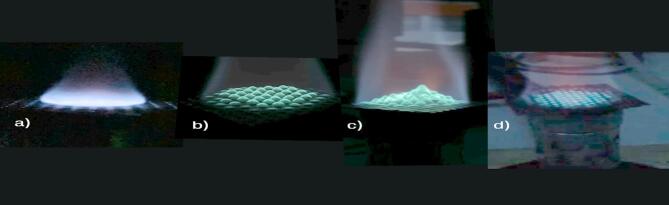
Flame behavior observed in a 2 mm channel under varying operating conditions.

Figure 6 presents a series of images illustrating flame behavior in a 2 mm channel under varying conditions. All experiments were carried out at an operating pressure of 1016 hPa, an ambient temperature of 22 °C, and a gas temperature of 20 °C.

In Fig. 6a, a mixture with 15% hydrogen content burns at a low exit velocity corresponding to throttled burner operation near the minimum load limit. Under such conditions, similar to those occurring during burner shutdown in domestic boilers, the flame becomes unstable and may be extinguished or break up. Figure 6b shows a stable flame with the same 15% hydrogen content at a low exit velocity representative of burner operation at minimum stable load (minimum power output), which corresponds well to part-load operation of atmospheric gas boilers. In Fig. 6c, the flame burns with 15% hydrogen at a normal exit velocity corresponding to nominal burner load. This case reflects standard boiler operation at rated capacity, where incomplete mixing inside the burner body leads to a partially diffusion-controlled flame with an elongated tip along the central axis. Finally, Fig. 6d illustrates combustion of a mixture with 30% hydrogen content at a low exit velocity, where the burning velocity nearly matches the exit velocity, causing the flame to stabilize directly within the mesh channels. Although hydrogen fractions above 25% were not included in the numerical analysis presented in this study, this case demonstrates that the flame-arresting principle of the mesh remains effective even under more demanding conditions, thereby confirming the safety margin of such burner designs.

**Fig. 7:**
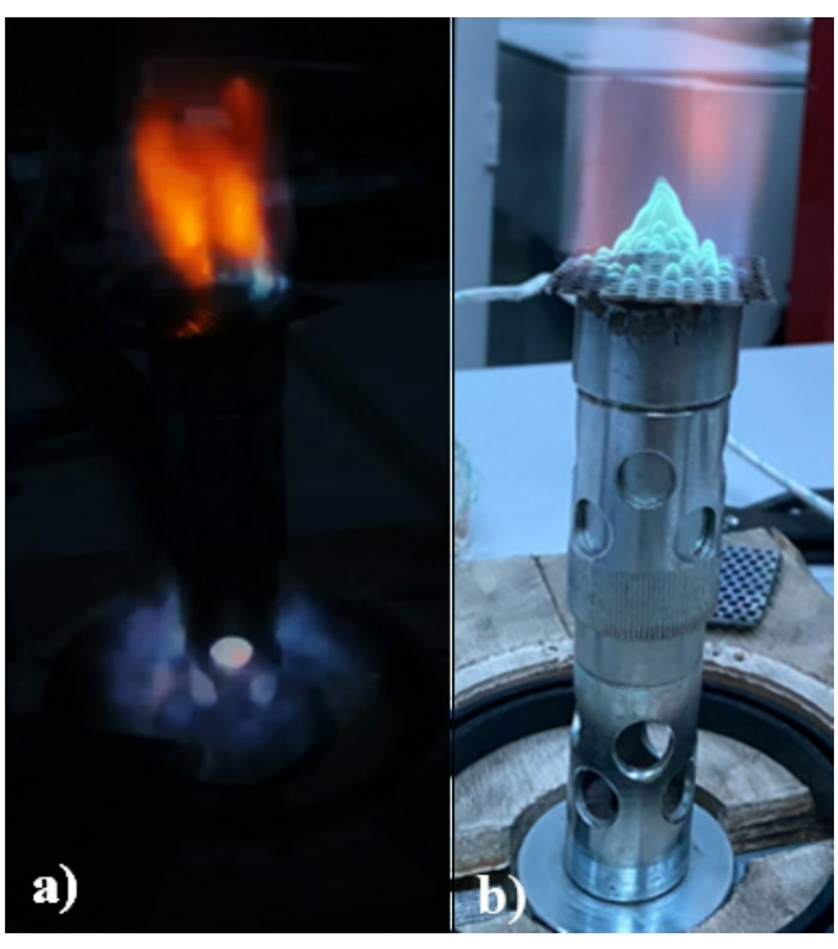
Photographic comparison of flame flashback (**a**), and normal flame (**b**) in a burner.

In Fig. 7a, flame flashback into the burner body is evident. This phenomenon occurs when the exit velocity of the fuel mixture from the burner is lower than the flame propagation speed. This effect is particularly common during performance regulation to minimum output, where the fuel supply is reduced to such a low pressure that the exit mixture does not reach the required velocity. In contrast, Fig. 7b shows a stable flame under normal operating conditions.

Modern condensing boilers on the European market are designed to operate with a natural gas–hydrogen blend containing up to 20% hydrogen and are equipped with burners fitted with meshes or slots of such dimensions that they prevent flame flashback and protect the combustion equipment from damage. On our market, the blending of green hydrogen is planned, and the carbon footprint will also be reduced by blending biomethane into natural gas. Biomethane differs only insignificantly from natural gas in its properties.

## Discussion and conclusion

This research examines the impact of hydrogen blending on the laminar flame speed of natural gas, with particular relevance to boiler systems. The results show that hydrogen addition increases the laminar flame speed, with a 25% hydrogen concentration leading to an 11% increase under stoichiometric conditions (λ = 1). With higher hydrogen concentrations, the maximum flame speed occurs at mixtures containing more fuel (lower λ values). Notably, at λ = 0.7, a 25% hydrogen concentration boosts flame velocity by 52%. Lambda significantly affects natural gas burner performance, with variations in λ influencing laminar flame speed, especially at higher hydrogen concentrations. These findings are consistent with Hu et al., who observed similar patterns in methane-hydrogen-air mixtures, and with model projections from Tommaso and Vassall for methane-hydrogen fuels^11,27^.

This study, therefore, explored a practical and cost-effective solution for improving the flashback resistance of existing small-scale burners by introducing a fine mesh with a precisely defined cell size. The mesh was installed at the burner outlet to ensure that the flame stabilized above the screen without penetrating the combustion chamber. For industrial-scale applications, the same design rule applies, i.e., a mesh with the minimum pore size corresponding to the hydrogen concentration must be installed. The approach is conceptually similar to that of Pers et al., who utilized a slotted metal plate, investigated how thermal quenching influences flashback phenomena in laminar hydrogen-enriched flames within a slot burner^28^. In that study, it was found that heat losses to the burner walls significantly affect the mechanisms behind flame flashback. Specifically, thermal quenching – where heat is lost from the flame to the surrounding material – can alter the propagation characteristics of the flame. In our study, the featured mesh had cell dimensions smaller than the critical quenching distance, thereby reliably suppressing flame propagation.

Such a retrofit can be performed during routine annual servicing by certified technicians, requiring no structural modifications to the boiler and taking only 5 to 10 min. The estimated cost of materials and labor is approximately €20–30, depending on boiler size. The condition of the mesh should be monitored during annual maintenance, with replacement carried out as necessary to account for thermal degradation.

While not directly examined in this study, operating under fuel-rich conditions with hydrogen may result in elevated NO_x_ emissions, an important consideration for modern boiler system design^29^. The increase in NO_x_ concentration in the flue gas is caused by an increase in flame temperature, but this study only addresses the issue of safety during the combustion of a hydrogen-natural gas mixture. In our experimental measurements, it was observed that the effect of hydrogen on NO_x_ emissions is not significant. The slight increase occurs due to the shift of the flame combustion from a rich mixture to a flame combustion under stoichiometric conditions, which is manifested by an increase in the temperature in the flame core and therefore a greater formation of thermal NO_x_. The issue of the increase in NO_x_ emissions of existing small heat sources in the future transition to such a fuel is currently neither legislatively resolved nor was it addressed in the initial experimental operations. Additionally, systems must adapt to fluctuating hydrogen concentrations as supply conditions change, influenced by variations in the blend composition provided by gas suppliers^22^.

The study also highlights the effects of hydrogen blending on flame flashback and quenching distance. Hydrogen enrichment increases the likelihood of flashback, as evidenced by Zhao et al., who reported flashback at 20% hydrogen in cooking burners and gas ovens during power regulation^23^. Similarly, Lo Basso et al. noted an increased risk of flashback in condensing boilers with hydrogen blending^24^. These findings emphasize that even moderate hydrogen concentrations can significantly alter combustion dynamics, necessitating modifications to burner design.

The observed reduction in quenching distance aligns with previous work by Fukuda et al., who demonstrated that even small amounts of hydrogen in a methane-hydrogen mixture significantly decrease quenching distance, particularly in lean and stoichiometric mixtures^12^. Calculated quenching distances in this study indicate that as hydrogen concentration increases, the characteristic channel dimension required to quench the flame decreases. This effect is most pronounced in lean mixtures (λ = 1.6), where a 36% reduction in quenching distance was observed at 23.1% hydrogen content. These results are further supported by Jung et al., who showed that hydrogen-air flames have reduced quenching distances due to enhanced reaction kinetics^14^.

While the presented study offers valuable insights into the combustion dynamics of hydrogen-enriched natural gas, it is limited to atmospheric pressure and a single flame configuration. In real burner systems turbulent or dynamic mixing can further enhance fuel–oxidizer interaction, increasing flame propagation rates and potentially amplifying the risk of flashback when hydrogen is present. Future investigations should include the effects of increased pressure, turbulence, and burner geometry on flashback and flame stability. The implications for boiler applications are considerable, as an increase in flame speed may necessitate design modifications to prevent flashback while maintaining stable combustion^30^. Furthermore, the shift of peak flame speed toward richer mixtures suggests that advanced combustion control mechanisms may be required to optimize combustion efficiency and emissions under varying hydrogen concentrations^31^.

One of the motivations for addressing burner modifications in this study stems from the widespread presence of older combustion systems that were not originally designed to accommodate hydrogen–natural gas mixtures. The increased flammability and explosion risk associated with hydrogen-enriched fuel blends pose a safety concern for such legacy equipment, particularly due to the heightened chance of flame flashback. Without appropriate adaptation, these systems may experience operational failures or, in the worst case, severe damage and safety hazards.

Experimental verification with a Mekker-Fischer type laboratory burner confirmed the calculated quenching distances, validating the theoretical model. The study also found that quenching distance is largely independent of screen thickness and material, with a 1.50 mm characteristic channel dimension proven sufficient to prevent flame flashback at hydrogen concentrations up to 40%.

Another key aspect of this study is the impact of hydrogen on the gross calorific value of the fuel mixture. Results show that higher hydrogen concentrations lower energy content per unit volume, aligning with other previous research^16^. For instance, at 20% hydrogen, the gross calorific value decreases by 14%, which could affect boiler efficiency and may require recalibration of combustion control systems to account for the lower energy density of hydrogen-enriched natural gas.

These findings highlight both the advantages and challenges of using hydrogen-enriched natural gas in decarbonizing boiler systems. While increasing hydrogen content can improve combustion performance and reduce carbon emissions, careful consideration of system design and operational factors is necessary to ensure safety and efficiency. Further research should focus on the effects of higher pressures and temperatures, relevant to industrial boiler applications, and explore hydrogen’s role in combustion stability, emissions, and heat transfer.

In conclusion, this study provides valuable insights into the combustion of hydrogen-enhanced natural gas, informing the development of optimized blending strategies for boilers. As the energy sector shifts toward lower-carbon solutions, this research contributes to ensuring a smooth and effective transition. These findings may also be extended beyond domestic boilers, providing a reference for designers of industrial burners, micro-turbines, and fuel-flexible combustion systems seeking to integrate variable hydrogen blends without compromising safety or efficiency. Moreover, it highlights the potential of computational tools for modeling larger clusters, enabling a broader assessment of hydrogen’s effects on diverse natural gas compositions beyond simple binary blends.

## Data Availability

The data that support the findings of this study are available from the corresponding author upon reasonable request.
